# Cobalt chloride has beneficial effects across species through a hormetic mechanism

**DOI:** 10.3389/fcell.2022.986835

**Published:** 2022-10-25

**Authors:** Alfonso Schiavi, Alessandra Runci, Teresa Maiorino, Francesco Davide Naso, Marta Barenys, Ellen Fritsche, Flavie Strappazzon, Natascia Ventura

**Affiliations:** ^1^ Institute of Clinical Chemistry and Laboratory Diagnostic, Medical Faculty, Heinrich Heine University, Düsseldorf, Germany; ^2^ Leibniz Research Institute for Environmental Medicine (IUF), Düsseldorf, Germany; ^3^ IRCCS Santa Lucia Foundation, Rome, Italy; ^4^ Institut NeuroMyogène, CNRS UMR5261—INSERM U1315, Université Claude Bernard Lyon1, Lyon, France

**Keywords:** aging, *C. elegans*, cobalt chloride, iron, healthspan, hormesis, neurodegeneration

## Abstract

Severe oxygen and iron deficiencies have evolutionarily conserved detrimental effects, leading to pathologies in mammals and developmental arrest as well as neuromuscular degeneration in the nematode *Caenorhabditis elegans.* Yet, similar to the beneficial effects of mild hypoxia, non-toxic levels of iron depletion, achieved with the iron chelator bipyridine or through frataxin silencing, extend *C. elegans* lifespan through hypoxia-like induction of mitophagy. While the positive health outcomes of hypoxia preconditioning are evident, its practical application is rather challenging. Here, we thus test the potential beneficial effects of non-toxic, preconditioning interventions acting on iron instead of oxygen availability. We find that limiting iron availability through the iron competing agent cobalt chloride has evolutionarily conserved dose-dependent beneficial effects: while high doses of cobalt chloride have toxic effects in mammalian cells, iPS-derived neurospheres, and in *C. elegans*, sub-lethal doses protect against hypoxia- or cobalt chloride-induced death in mammalian cells and extend lifespan and delay age-associated neuromuscular alterations in *C. elegans*. The beneficial effects of cobalt chloride are accompanied by the activation of protective mitochondrial stress response pathways.

## Introduction

Severe hypoxia has evolutionarily conserved and widely described deleterious consequences, ranging from ischemic stroke and myocardial infarction in mammals to developmental arrest and neuromuscular degeneration in the nematode *Caenorhabditis elegans* (*C. elegans*) (Rodriguez et al., 2013; Lee et al., 2019). In normoxic conditions, HIF1α (hypoxia-inducible-factor) is hydroxylated by enzymes that require O_2_, Fe^++^, and 2-oxoglutarate: at proline by PHD1-3 (prolyl-4-hydroxylase domain-containing enzymes) or at asparagine by FIH (factor inhibiting HIF). This leads to the subsequent ubiquitination and degradation of HIF1α via the proteasome. Low oxygen and iron concentrations diminish PHD and FIH hydroxylase activities and prevent HIF1α degradation. This favors HIF1α translocation into the nucleus and binding to co-activators, which ultimately induces its transcriptional activity resulting in the modulation of a variety of downstream genes containing hypoxia response elements (HRE) (Wenger, 2002; Powell-Coffman, 2010). Accordingly, iron chelators such as deferoxamine (DFO) or bipyridine (BP), and iron-competing metals such as cobalt chloride (CoCl_2_) or nickel chloride (NiCl_2_) have been shown to work as hypoxia mimetics by deactivating the hydroxylases (Goldberg et al., 1988). Furthermore, similar to severe hypoxia, severe iron depletion is also involved in the pathogenesis of different human disorders and it leads to developmental arrest in *C. elegans* (Schiavi et al., 2015; Schiavi et al., 2020). Iron is an essential factor for several enzymes and cellular reactions, and therefore, its homeostasis must be finely tuned. Not only iron deprivation but also its excessive accumulation has evolutionarily conserved detrimental effects. Iron overload concurs to the pathogenesis of age-associated neuronal pathologies such as Parkinson’s or Alzheimer’s diseases, and it is a typical hallmark of the aging process, thereby shortening lifespan in *C. elegans* (Lee et al., 2006; Schiavi et al., 2015).

Friedreich’s ataxia (FRDA) is the most frequently inherited recessive ataxia, and it is ascribed to severe deficiency of frataxin, a nuclear-encoded mitochondrial protein involved in iron–sulfur cluster (ISC) protein biogenesis and iron homeostasis. Accumulation of iron within mitochondria, with consequent alteration of ISC formation and induction of a cytosolic iron-starvation response, has been recognized as a consequence of frataxin deficiency involved in disease pathogenesis (Anzovino et al., 2014). In line with the disease state, and similar to severe oxygen and iron deprivation, severe or complete frataxin deficiency also displays evolutionarily conserved detrimental effects, leading to lethality in mice and to developmental arrest in *C. elegans* (Puccio et al., 2001; Ventura et al., 2005). Nonetheless, non-toxic levels of iron depletion, achieved with the iron chelator BP or through frataxin (*frh-1*) silencing, extend *C. elegans* lifespan through hypoxia-like induction of mitophagy (Schiavi et al., 2015). Moreover, exposure to non-lethal levels of hypoxia (or hypoxia preconditioning, HP) has been shown to prevent the detrimental effects of severe hypoxia-induced neuronal degeneration in an evolutionarily conserved manner (Dasgupta et al., 2007; Liu et al., 2021). While the potential beneficial health effects of HP are evident, its actual exploitation is hampered by the low feasibility of its practical application. In this study, we thus asked whether non-toxic, preconditioning interventions acting on iron instead of oxygen availability may provide beneficial effects too. We found that similar to HP, limiting iron availability through CoCl_2_ treatment has evolutionarily conserved beneficial effects, protecting against severe hypoxia- and age-induced neuromuscular alterations. We thus provide evidence that limiting iron availability represents a potential feasible strategy to delay aging and associated neuromuscular pathologies possibly *via* autophagy/mitophagy induction.

## Results

### Cobalt chloride promotes healthspan in *C. elegans*

**FIGURE 1:**
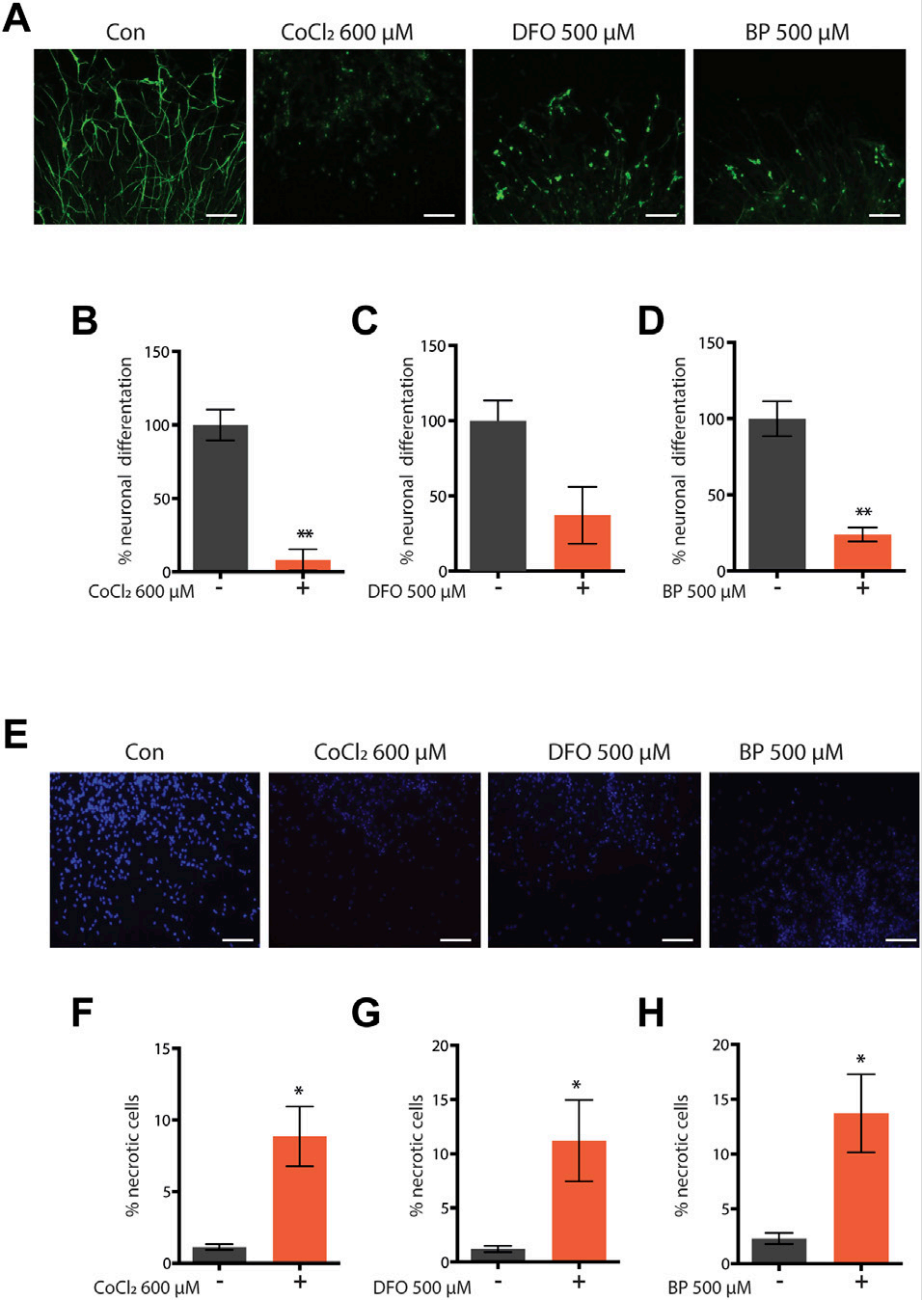
**(A–H)** Representative pictures and quantification of neuronal differentiation **(A–D)** and necrotic cell death **(E–H)** of mouse embryo iPS-derived neurospheres left untreated or treated with cobalt chloride 600 µM (CoCl—600 μM) **(A–F)** for 48 h, deferoxamine 500 µM (DFO 500 μM) **(C,G)** for 72 h, or 2,2′-dipyridyl 500 µM (BP 500 μM) **(D,F)** for 72 h during neuronal precursor cells (NPC) differentiation. Bar graph represents means ± SEM from *n* = 3 independent replicas where N = 5 randomly chosen fields were scored. Unpaired t-test, *p value < 0.05 and **p value < 0.01 versus untreated. Above the bar graphs are shown the representative fluorescence pictures of the corresponding conditions. Scale bar are 100 µm. Green fluorescence pictures show neurons stained with beta-III-tubulin **(A)**, and blue fluorescence pictures show nuclei stained with Hoechst 33342 **(E)**.

Iron is a vital cofactor for numerous proteins and enzymatic reactions and its intracellular concentration has to be finely tuned for appropriate organism homeostasis (Schiavi et al., 2020). Accordingly, both excess iron accumulation and depletion have toxic effects across species. Interestingly, we have previously shown that contrary to its severe deficiency, sub-lethal iron depletion through the iron chelator BP or through *frh-1* silencing extends *C. elegans* lifespan (Schiavi et al., 2015). Limiting iron availability acts as a hypoxia mimetic, and exploiting hypoxia mimetics to prevent or protect against hypoxia- or age-induced neuromuscular pathologies may provide different advantages over hypoxia preconditioning. We thus sought to investigate the beneficial effects of additional modulators of iron availability. Consistent with the detrimental role of severe iron depletion especially during development, we found that the iron chelators DFO and BP as well as the iron-competing metal CoCl_2_ significantly reduce neuronal differentiation (Figures 1A–D) and viability (Figures 1E–H) in mouse neural progenitor cell (NPC)-derived neurospheres, a very sensitive and powerful system for developmental neurotoxicity studies (Fritsche et al., 2018).

**FIGURE 2:**
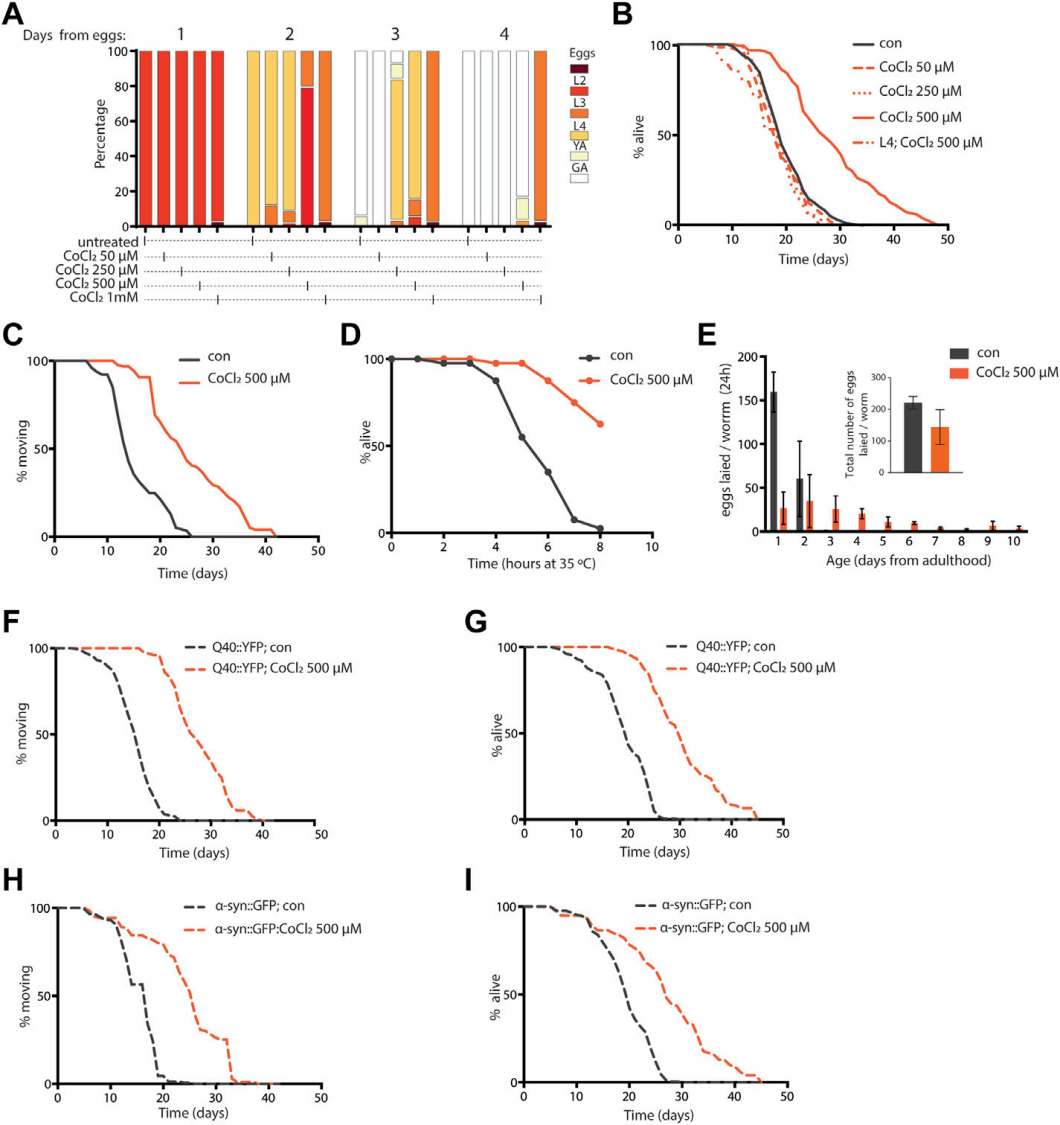
**(A)** Developmental rate of wild-type animals—from eggs through four different larval stages, until young (YA), and finally gravid (GA) adults—either left untreated or treated from eggs with 50 µM, 250 µM, 500 µM, and 1 mM cobalt chloride (CoCl_2_ 50 µM, CoCl_2_ 250 µM, CoCl_2_ 500 µM, and CoCl_2_ 1 mM), and followed for 4 days after hatching at 20°C. **(B)** Kaplan–Meier survival curves at 20°C of wild-type worms, left untreated (con) or treated from eggs with 50 µM, 250 µM, and 500 µM CoCl_2_ and starting from L4 larval stage with 500 µM CoCl_2_. Comparison between curves was performed using the log‐rank test (Table 1). (**C,D)** Animals’ locomotion activity **(C)** and survival against heat shock **(D)** of wild-type strain left untreated (con) or treated with 500 µM cobalt chloride (CoCl_2_ 500 µM). Comparison between curves was performed using the log-rank test. **(E)** Fertility and brood size over time of wild-type worms treated as in (C). **(F–I)** Animal locomotion activity **(F,H)** and survival against heat shock **(G,I)** of *unc-54p*::Q40::YFP **(F,G)** or *unc-54p*::a-syn::YFP **(H,I)** strain left untreated (con) or treated with 500 µM cobalt chloride (CoCl_2_ 500 µM). Comparison between curves was performed using the log-rank test.

Similar to complete *frh-1* deficiency (Ventura et al., 2005) or high doses of BP (Schiavi et al., 2015), a high concentration of CoCl_2_ also arrested development in *C. elegans* (Figure 2A). Most notably, we found that non-toxic doses of CoCl_2_ extend lifespan and healthspan (motility period) in a stage- and dose-dependent manner (Figures 2B,C; Table 1). This is in line with what has been previously observed with BP treatment or depletion of different mitochondrial electron transport chain (ETC) regulatory proteins including *frh-1* (Dillin et al., 2002; Rea et al., 2007; Schiavi et al., 2015). Pro-longevity doses of CoCl_2_ also significantly reduced the animals’ size (Supplementary Figure S1), increased their resistance to heat shock (Figure 2D), and extended their fertility period without modifying the overall brood size (Figure 2E). These are all parameters also associated with mitochondrial-stress extension of lifespan.

**TABLE 1:** Summary of lifespan analysis.

Genotype	Treatment	Mean lifespan (days)	Standard error	*p* vs. con	Age at 100% mortality (days)	Total/censor	N
Wild-type (N2)	Con	19.8	0.4		34	275/22	4
	CoCl_2_ 500 µM	28.6	0.7	<0.0001	48	195/41	3
	CoCl_2_ 250 µM	19.6	0.4	0.9	28	145/19	2
	CoCl_2_ 50 µM	20.3	0.7	1	30	145/47	2
	CoCl_2_ 500 µM/L4	17.6	0.9	0.9	26	130/97	2
AM141 (*unc-54p*::Q40::YFP)	Con	19.5	0.4		30	195/24	3
	CoCl_2_ 500 µM	30.6	0.6	<0.0001	45	195/76	3
NL5901 (unc-54p::αsyn::YFP)	Con	19.7	0.4		30	195/22	3
	CoCl_2_ 500 µM	27.4	0.8	<0.0001	45	195/86	3

Excess iron is a typical hallmark of the aging process which concurs to the pathogenesis of age-associated neurodegenerative diseases, and progressive accumulation and toxicity of aggregation-prone proteins in these diseases are worsened by excess iron levels (Lee and Andersen, 2006; Schiavi et al., 2020). We thus wondered whether CoCl_2_ could promote beneficial effects in *C. elegans* strains expressing human aggregation-prone proteins, poly-Q40 and α-synuclein (under muscle-specific promote, *unc-54*), and widely exploited as model systems to respectively study Huntington’s and Parkinson’s diseases (Alexander et al., 2014; Caldwell et al., 2020; Liang et al., 2020). Notably, CoCl_2_ also significantly extended lifespan and locomotion ability in the Huntington’s and Parkinson’s disease models (Figures 2F–I). Moreover, we observed an age-dependent accumulation of poly-Q40 and α-synuclein aggregates, which was significantly reduced by pro-longevity CoCl_2_ treatment (Supplementary Figure S1). Thus, in *C. elegans*, CoCl_2_ extends health- and lifespan in a wild-type background as well as in age-associated disease models.

### Cobalt chloride activates mitochondrial stress response pathways in *C. elegans*

**FIGURE 3:**
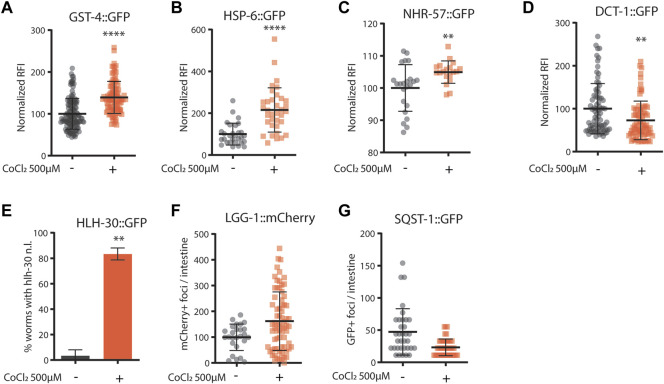
**(A–G)** Quantification of fluorescence reported strains in adult worms (4 days old) left untreated (−) or treated from eggs with CoCl_2_ 500 µM (+). Relative fluorescence intensity normalized to animals’ size was quantified in GST-4::GFP **(A)**, HSP-6::GFP **(B)**, NHR-57::GFP **(C)**, and DCT-1::GFP **(D)**. Percentage of worms with HLH-30::GFP nuclear localization **(E)**. Fluorescence foci number was quantified in LGG-1: mCherry **(F)** and SQST-1::GFP **(G)**. Bar graph represents means ± SD (*n* = 3, N = 10–15); lines in the scatter plots represent means ± SD (*n* = 3, N = 10–15). Comparison between con and CoCl_2_ was performed using the unpaired t-test. **p value < 0.01 and **** *p* value < 0.0001, versus con.

Given the striking similarity between animals’ phenotypes upon CoCl_2_ treatment and pro-longevity mitochondrial stress (Rea et al., 2007; Ventura et al., 2009), we then wondered whether the beneficial effects of CoCl_2_ preconditioning in *C. elegans* could be ascribed to the induction of mitochondrial stress response signaling. Interestingly, like *frh-1* silencing (Ventura et al., 2009; Schiavi et al., 2015), pro-longevity doses of CoCl_2_ in *C. elegans* significantly increased the expression of genes normally induced by mitochondrial stress such as *gst-4* (glutathione-S-transferase), *hsp-6* (mitochondrial unfolded protein response), and *nhr-57* (hypoxia–inducible gene) and reduced the expression of the mitophagy marker *dct-1*/BNIP3 (Figures 3A–D). CoCl_2_ also strongly induced the nuclear translocation of the autophagy regulator transcription factor *hlh-30*/TFEB, but only marginally affected the number of *lgg-1*/LC3 and of *sqst-1*/p62 foci, the other two central autophagy regulatory genes (Figures 3E–G). These changes suggest CoCl_2_ treatment induces mitochondrial stress that activates the autophagic machinery.

In mammals, Bnip3/*dct-1*-regulated mitochondrial autophagy is induced in response to hypoxia in a HIF-1-dependent manner (Zhang et al., 2008; Bellot et al., 2009), and in *C. elegans*, *frh-1* silencing extends lifespan *via* mitophagy activation and in a *hif-1*-dependent manner (Schiavi et al., 2015). Here, we find that the *C. elegans* HIF-1 homolog, *hif-1*, is required for the survival of CoCl_2_-treated animals. Indeed, while *C. elegans* wild-type animals treated with pro-longevity doses of CoCl_2_ reached the fertile stage slower than untreated animals (Figure 2A), *hif-1* mutants completely arrested their development upon CoCl_2_ treatment (Supplementary Figure S2). Thus, CoCl_2_ activates a mitochondrial stress response likely in a *hif-1*-dependent manner.

### Cobalt chloride preconditioning has beneficial effects across species

**FIGURE 4:**
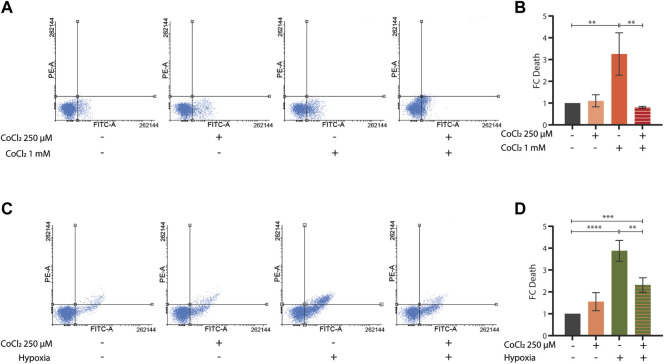
**(A–D)** Representative FACS plot **(A,C)** and quantification **(B,D)** of cell death assays performed in HeLa cells left untreated or treated with CoCl_2_ 1 mM for 48 h **(A,B)** or severe hypoxia (<1% O_2_ for 48 h) **(C,D)** alone or in combination with either a CoCl_2_ 250 µM pre-treatment for 24 h followed by 4 h recovery **(A,B)** or a CoCl_2_ 250 µM co-treatment for 48 h, respectively. Bar graph represents means ± SD (*n* = 3); two-way ANOVA (Tukey’s multiple comparison test). **p value < 0.01 and **** *p* value < 0.0001.

To further investigate the potential protective effects of CoCl_2_ preconditioning, we then turned to mammalian cells. As expected, treatments with either high doses of CoCl_2_ (1 mM for 48 h) or severe hypoxia (<0, 1% O_2_ for 48 h) induced apoptosis in HeLa cells (Figures 4A,C). Notably, milder doses of CoCl_2_ provided protection against apoptosis either when applied as a pretreatment (250 uM for 24 h, followed by a recovery period of 4 h) before exposure to higher doses of CoCl_2_ (Figures 4A,B) or when co-applied (250 uM for 48 h) during severe hypoxia treatment (Figures 4C,D).

**FIGURE 5:**
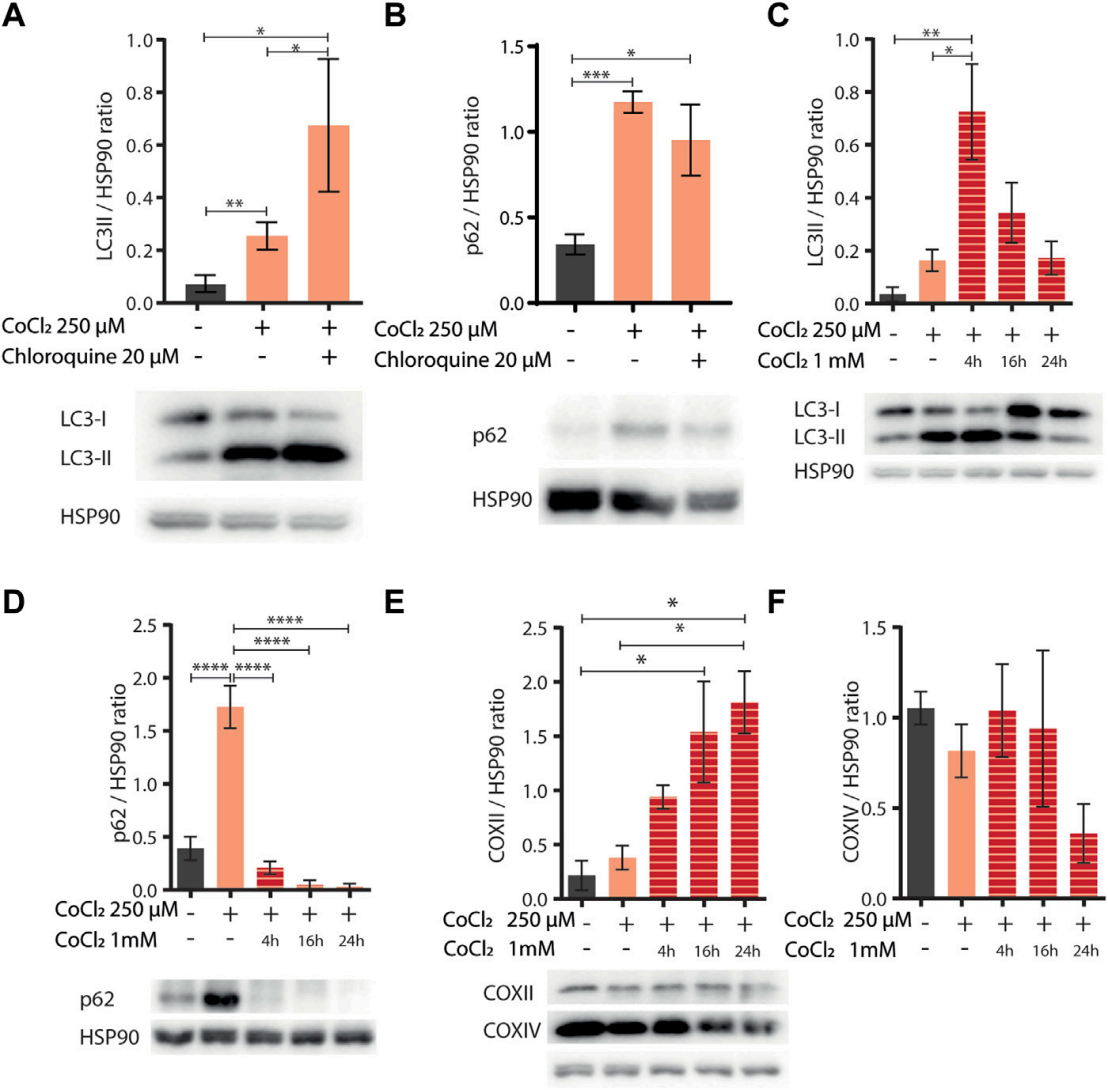
**(A,B)** Western blot analysis of LC3II **(A)** and p62 **(B)** proteins extracted from HeLa cells left untreated (−) or treated (+) with 250 µM of CoCl_2_ alone or in combination with 20 µM of chloroquine. Proteins were normalized using HSP90; below the bar graph are shown the representatives Western blot scan. Bar graphs represent means ± SD (N = 3), two-way ANOVA (Tukey’s multiple comparison test). *p value < 0.05 and **p value < 0.01. **(C–F)** Western blot analysis of LC3II **(C)**, p62 **(D)**, and COXII **(E)** and COXIV **(F)** proteins extracted from HeLa cells left untreated (−) or treated (+) with 250 µM of CoCl_2_ alone or in combination with 1 mM of CoCl_2_ at different exposure time 4, 16, and 24 h. Proteins were normalized using HSP90, and below the bar graph **(C–E)** are shown the representatives Western blot scan. Bar graph represents means ± SD (N = 3), two-way ANOVA (Tukey’s multiple comparison test). *p value < 0.05, **p value < 0.01, ***p value < 0.001, and ****p value < 0.0001.

Induction of autophagy and/or mitophagy is an attractive common determinant underlying the protection against aging and hypoxia induced by CoCl_2_ preconditioning across species. Indeed, these fundamental cellular turnover processes are required to protect cells against hypoxia-induced cell death (Zhang et al., 2008) or against proteotoxicity (Florez-McClure et al., 2007; Guerrero-Gomez et al., 2019) and to extend *C. elegans* lifespan upon other iron depleting interventions such as *frh-1* RNAi or the iron chelator BP (Schiavi et al., 2013; Schiavi et al., 2015). Thus, to assess the effect of CoCl_2_ preconditioning on autophagy activity, we analyzed the LC3II status [a well-known autophagosome marker (Klionsky et al., 2016)] by Western blot in mammalian cells, at early time points (before apoptotic cell death was observed), upon mild doses of CoCl_2_ either alone or in combination with chloroquine [CQ, an inhibitor of the fusion between autophagosomes and lysosomes used to ascertain the integrity of the autophagic flux (Klionsky et al., 2016)]. Mild doses of CoCl_2_ significantly increased LC3II protein levels, which was further increased in the presence of CQ, thus clearly supporting the induction of intact autophagy flux by CoCl_2_ preconditioning (Figure 5A). We then wondered whether mild doses of CoCl_2_ also modulate the expression of p62/SQSTM1 protein, a targeted receptor for autophagic degradation of ubiquitinated substrates, which is often used as a reporter of autophagy activity (Klionsky et al., 2016). Surprisingly, LC3II accumulation upon CoCl_2_ preconditioning was paralleled by a chloroquine-insensitive induction of p62 (Figure 5B). This indicates that the autophagic flux induced by CoCl_2_ preconditioning is not sufficient to decrease the high levels of expression of p62 likely ascribed to a strong increase in its transcriptional expression and/or stability. Consistent with this possibility, exposure of preconditioned cells to higher doses of CoCl_2_ (1 mM) significantly increased LC3 turnover at early time points and decreased p62 expression, indicating a strong induction of autophagy compared to CoCl_2_ preconditioning alone (Figures 5C,D). The expressions of COXII and COXIV, two classical markers to reveal mitochondrial turnover (Klionsky et al., 2021), were instead inconsistently modulated (Figures 5E,F). To ascertain the effect of CoCl_2_ preconditioning in prompting autophagy, we then compared the effect of 1mM CoCl_2_ in HeLa cells pre-exposed or not to milder doses of CoCl_2_. Our data confirmed that the preconditioning favors LC3II conversion compared to cells treated only with 1 mM CoCl2 as well as a stronger decrease of p62 (Supplementary Figures S3A,B). Moreover, we noticed that 1 mM CoCl2 alone stimulates the production of COXII and COXIV after 16 h of treatment (Supplementary Figures S3C,D). Notably, at this early time point used to analyze autophagy, we could already observe morphological changes hinting CoCl_2_ preconditioning-induced protection in cells treated with 1 mM of CoCl_2_ compared to cells undergoing only the severe treatment (Supplementary Figure S4). Thus, according to a hormetic paradigm, CoCl_2_ preconditioning promotes healthspan likely *via* its hypoxia mimetic activity (compete with iron in HIF-1 activation), and autophagy induction may represent its underlying mechanism, which is in common with other treatments which limit iron availability (i.e., *frh-1* RNAi, BP).

## Discussion

Overall, our work provides evidence that CoCl_2_ preconditioning displayed evolutionarily conserved beneficial effects: it protects *C. elegans* against age- and proteotoxicity-induced neuromuscular damage and functional decline as well as mammalian cells against hypoxia-induced death. While we showed in the past that reducing the expression of the mitochondrial protein FRH-1 (*via frh-1* silencing) extends lifespan through an iron starvation response and in a *hif-1-*dependent manner, whether CoCl_2_ promotes health span *via hif-1*-activation (through its iron competing effect) and/or also by inducing mitochondrial stress remains to be evaluated. Nonetheless, the ultimate effect of reducing iron availability is an improvement in health span, and this has important repercussions in light of the emerging role of hypoxia preconditioning in preventing neuromuscular deficits. Indeed, while the potential therapeutic benefits of hypoxia preconditioning are evident and have been suggested (Li et al., 2017; Caricati-Neto et al., 2019), fine-tuning oxygen levels to trigger beneficial effects without surpassing the critical threshold is not trivial and has low practical applications. Thus, the identification of more finely tunable interventions mimicking hypoxia, such as iron chelators already used in the clinic or undergoing clinical trials (Wijesinghe et al., 2021; Yang et al., 2021; Reddy et al., 2022) or modulators of downstream molecular players mediating their beneficial effects, may suggest more practicable strategies to interfere with the detrimental features associated with aging and severe hypoxia. Different phenotypic features are concurrently associated with the pro-longevity effect induced by mild doses of CoCl_2_ such as reduced animals’ size and egg-lay rate, prolonged fertility period, and increased resistance to stress (Ventura et al., 2005; Rea et al., 2007) and delayed neuromuscular decline during aging (Maglioni et al., 2014), which strikingly resemble those induced by mild mitochondrial stress. Notably, we showed that limiting iron availability with CoCl_2_ displays beneficial effects only when applied at non-toxic doses. This threshold effect has been previously demonstrated for HP, mitochondria hormesis (e.g., with *frh-1* silencing), and the iron chelator BP, which only display beneficial properties at mild doses (Rea et al., 2007; Schiavi et al., 2015; Merry and Ristow, 2016; Li et al., 2017; Caricati-Neto et al., 2019). While HP has known beneficial effects against severe hypoxia-induced neuromuscular damage across species (Dasgupta et al., 2007), we show here that mild treatment with CoCl_2_ may also similarly provide protection against severe hypoxia, aging, or accumulation of proteotoxic proteins. It is worth noting that the beneficial effect of the different iron depleting agents is dose-dependent and similar to mitochondrial stress (Dillin et al., 2002; Rea et al., 2007), it is elicited only when the agents are applied at sub-lethal doses starting during animal development. This clearly indicates that mitochondria preconditioning-associated metabolic remodeling must occur early in life to promote protection against aging and age-associated features (Maglioni et al., 2019).

In search of a common molecular mechanism mediating the beneficial effects of the different iron modulating interventions, we initially considered *hif-1*-regulated autophagy or mitophagy. Indeed, autophagy protects against apoptosis (Marino et al., 2014) and against the toxicity of aggregation-prone proteins during aging (Florez-McClure et al., 2007; Guerrero-Gomez et al., 2019). Autophagy is also induced by hypoxia and by frataxin silencing across species and it is required to mediate the pro-longevity effect of *frh-1*-depletion in *C. elegans* (Schiavi et al., 2013; Schiavi et al., 2015). Our results in *C. elegans* clearly suggest that CoCl_2_ acts in a *hif-1*-dependent manner. Moreover, the combined data in worms and cells support the induction of bulk autophagy as a likely downstream mediator of the beneficial effect of CoCl_2_ preconditioning, but whether this is activated in a *hif-1*-dependent manner, *via* mitochondrial stress responses or other mechanisms, remains to be systematically investigated. Very interestingly, our data suggest that low doses of CoCl_2_ “prepare” the cells for better autophagy activation by subsequent higher doses of hypoxia-like stress [which would otherwise have deleterious effects on the cells (Pourpirali et al., 2015)]. This is a classical hormetic scenario (Cypser and Johnson, 2002; Gems and Partridge, 2008), and it will be important to establish whether and how CoCl_2_ preconditioning is also protective in other pathological or stressful contexts.

Paradoxically, our data point to a strong accumulation of p62 as a potential underlying mechanism of the CoCl_2_ preconditioning effect. While an increase p62 may indicate a block of autophagy flux, which in a more severe hypoxic scenario (higher doses of CoCl_2_) was linked to induction of apoptosis (Pourpirali et al., 2015), in our preconditioning paradigm, it could instead favor autophagy activation by additional stress. The overexpression of p62 by CoCl_2_ preconditioning could be ascribed on the one hand to its increased stability *via* different post-translational modifications, which might prevent its association with the E3 ligase Keap1 (Kelch-like ECH-associated protein 1) followed by poly-ubiquitination and degradation in the proteasome (Pan et al., 2016; Sun et al., 2016; Xu et al., 2019; Feng et al., 2021). On the other hand, overexpression of p62 could be induced by transcriptional activation for instance by the redox transcription factor Nrf2 (nuclear factor erythroid 2-related factor 2). Since Nrf2 is also a substrate for Keap1, the binding of p62 to Keap1 results in a positive feedback loop leading to Nrf2 activation (Jain et al., 2010; Komatsu et al., 2010). Notably, we showed that CoCl_2_ in *C. elegans* increases the expression of *gst-4* and of *dct-1*, two genes induced by mitochondrial stress in an Nrf2/*skn-1*-dependent manner (Ventura and Rea, 2007; Palikaras et al., 2015). While our combined findings in nematode and cells point to prompted activation of autophagy by CoCl_2_ preconditioning followed by more severe treatment, they do not unambiguously support the induction of mitophagy. These results may be ascribed by the masking effects of cell-specific or cell-non-autonomous processes but also to context-specific roles of p62. Indeed, p62 is not always required to mediate mitophagy (Narendra et al., 2010) and it may also modulate other forms of cell death such as ferroptosis [e.g., *via* Nrf2 activation (Sun et al., 2020; Chen et al., 2022; Tang et al., 2022)].

Interestingly, while *frh-1* silencing induces both autophagy and mitophagy, the Nrf2/*skn-1* pathway and extends lifespan in a *hif-1*-dependent manner, the iron chelator BP only induces mitochondrial but not bulk autophagy, induces the Nrf2/*skn-1* pathway, and extends lifespan in a *hif-1*-independent manner (Schiavi et al., 2013; Schiavi et al., 2015). Moreover, while CoCl_2_ modulates gene expression in a *hif-*1-dependent manner across species (Triantafyllou et al., 2006; Padmanabha et al., 2015), it can also induce hypoxia-regulated genes, such as *nhr-57*, in a *hif-1*-independent manner (Padmanabha et al., 2015). Thus, while reducing iron availability can mimic the beneficial effects of HP, the common underlying molecular mechanism of the different hypoxia-mimetics may in fact be through *hif-1*-independent but Nrf2-related pathways.

Further studies will be required to actually clarify whether autophagy, mitophagy, or other forms of iron-regulated processes such as ferritinophagy or ferroptosis are involved in the protective effects of CoCl_2_ or other interventions reducing iron availability. In conclusion, we identified CoCl_2_ as a new agent protecting against hypoxia- and age-induced neuromuscular degeneration, possibly *via hif-1-* and/or Nrf2-regulated pathways.

## Materials and methods

### Nematodes

#### *C. elegans* strains and culture conditions

The following strains were used in this study: N2 wild type, AM141: rmIs133 [*unc*-*54p*::Q40::YFP], NL5901: pkIs2386 [*unc*-*54p*::alpha-synuclein::YFP + *unc*-*119*(+)], CL2166: dvIs19[pAF15(*gst*-*4p*::GFP::NLS)], SJ4100: zcIs13[*hsp*-*6p*::GFP], ZG120: ials07[*nhr*-*57p*::GFP, *unc*-*119*(+)], JIN1679: jinEx10 [*hlh*-*30p*::*hlh*-*30*::GFP + *rol*-*6*(*su1006*)], vkEx1093: [*nhx*-*2p*::mCherry::lgg-1], HZ589: bpIs151[*sqst*-*1p*::SQST-1::GFP; unc-76(+)]IV; him-5(e1490)V, IR1431: N2;Ex001[*dct*-*1p*::DCT-1::GFP], ZG31: *hif*-*1*(*ia4*).

All strains were maintained and kept synchronized by egg lay at 20°C on nematode growth media (NGM) agar supplemented with *Escherichia coli* OP50 unless otherwise described.

#### Chemical treatments

Cobalt chloride (CoCl2 C8661, Sigma-Aldrich) was dissolved at 0.1 M stock solution in ddH_2_O, sterilized using a 0.22-μM filter, and supplemented to the NGM after autoclaving at the concentration of interest.

2,2′ dipyridyl (BP, 4153 Carl Roth) was dissolved in ddH_2_O and added to NGM to the indicated concentrations.

#### Lifespan

Survival analyses were carried out with the standard procedure in the field as routinely carried out in our laboratory (Torgovnick et al., 2018). Briefly, a synchronized population of 60–80 worms was used to start the lifespan analysis. Animals were transferred on fresh plates every day during the fertile phase and at the end of the fertile period, worms were transferred every other day. Animals not able to move upon prodding and with no pharyngeal pumping were scored as dead animals and were scored as not moving when no sinusoid locomotory activity was observed upon prodding. Survival analysis was performed in OASIS 2 using the Kaplan–Meier estimator. Statistical differences were evaluated using the log-rank test between the pooled population of worms, and *p* values were adjusted for multiple comparisons by the Bonferroni method.

#### Health span—movement

Movement was assessed in the same populations used for the lifespan assay. Worms able to crawl spontaneously or after manual stimulus were considered as moving, while dead worms or animals without crawling behavior were considered as not moving. Statistical analysis was performed as described for lifespan.

#### Fecundity

The fecundity of animals was estimated by counting the eggs laid in a period of 4 h; three worms on four different plates were used in each condition and repeated in three independent trials.

#### Heat shock resistance

Twenty wild-type animals for each condition were maintained from eggs at 20°C on plates with or without cobalt chloride 500 μM. On the first day of adulthood, they were moved to 35°C and the survival rate was counted every hour for 8 h.

#### Quantification of GFP-transgene expression by fluorescence microscopy

The nematodes were placed in a 15 µl S-Basal plus levamisole 10 mM drop on a microscope glass slide, covered with a cover slide, and immediately imaged. Pictures were acquired with an Imager 2 Zeiss fluorescence microscope, with magnification 25-fold. The images were analyzed with the software ImageJ (http://imagej.nih.gov/ij/). The bright field images were used to generate the mask to select each single worm pictured, and consequently to measure the fluorescence in the correspondent fluorescent filter.

#### Autophagy measurements

mCherry-positive foci from the *Pnhx-2*::mCherry::LGG-1 transgenic strain were counted in the intestine of young adult worms. To quantify p62::GFP puncta, the number of GFP foci was quantified in the anterior pharyngeal bulb of the *Psqst-1*:SQST-1::GFP transgenic strain. Worms were mounted on agarose pads, pictures were acquired using 100-fold magnification on a Zeiss Axio Imager 2 microscope, and the foci were analyzed with Fiji (Schindelin et al., 2012).

#### HLH-30 nuclear localization measurement

The number of worms showing HLH-30::GFP nuclear localization was visually counted using a Zeiss Axio Imager 2 (100-fold magnification). Worms were anesthetized using 10 mM levamisole in S-Basal and acquired within 5 min of mounting on glass slides. The percentage of worms with HLH-30 nuclear localization was calculated for each condition and plotted as mean ± SEM.

#### Poly-Q40 aggregation measurement

Poly-Q40 formations were acquired using a 25-fold magnification on a Zeiss Axio Imager 2 microscope. Worms were acquired at 4,7, and 10 days after hatching, and left untreated or treated from eggs with CoCl_2_ 500 µM. The number of poly-Q40 aggregates was counted using the “analyze particles…” tools in ImageJ after thresholding and converting the acquired images in a binary form. The number of poly-Q40 was counted per worm by manually selecting the single worms. Worms were anesthetized using 10 mM levamisole in S-Basal and acquired within 5 min.

### Mammalian systems

#### Cell line maintenance and treatment

The human cervix carcinoma (HeLa) cell line was used in this study. The cells were incubated at 37°C with 5% CO_2_ and maintained in DMEM with 1.0 g/L glucose (PAN-Biotech GmbH, Germany) as a standard medium, supplemented with fetal bovine serum (FBS, Sigma-Aldrich), 10% and Penicillin/Streptomycin/Amphotericin B Mix (PAN-Biotech GmbH, Germany), 1%.

For cobalt chloride (CoCl_2_ 15862, Sigma-Aldrich) treatment, it was directly added to the plates at the desired concentration. To generate the hypoxia status, the seeded cells were placed into a modular incubator chamber (Billups-Rothenberg). A gas mixture (95% N_2_ and 5% CO_2_) was flushed (15 l/min) into the chamber for 10 min and then incubated for the indicated time. After the different treatments, the cells were immediately collected for cell death detection.

Autophagosome–lysosome fusion was blocked with chloroquine (CQ) at 20 μM for 1 h (Sigma-Aldrich, United States). Images of HeLa cells after CoCl_2_ treatments were acquired using the ZOE Fluorescent Cell Imager (Bio-Rad, United States).

#### Cell death detection by FACS analysis

HeLa cells, untreated or treated with cobalt chloride and/or hypoxia, were washed with PBS and collected by centrifugation after trypsinization. The cell pellet was suspended in binding buffer (Annexin V-FITC kit; BioVision, Milpitas, CA, United States), followed by the addition of 5 μl Annexin V-labeling solution (BioVision) and 5 μl propidium iodide (BioVision). Cells were then incubated for 5 min at room temperature and analyzed *via* fluorescence-activated cell sorting (FACS; BD FACSCanto II).

#### Autophagy detection by Western blotting

HeLa cells were lysed in RIPA buffer (150 mM NaCl, 50 mM Tris with pH 7.4, 1% Triton, 0.5% Nonidet P40, 10% glycerol, and 2.5% sodium deoxycholate) plus protease and phosphatase inhibitors (Roche Diagnostic, Germany, 11836153001). Protein concentrations were determined with the Bio-Rad Protein Assay Dye kit (Bio-Rad, United States, 5000006). Cell extracts were resolved by SDS-PAGE and transferred onto PVDF membranes (Millipore, Germany, Immobilon-P IPVH00010). Blocking was performed at room temperature in TBS 1X 0.1% Tween 5% low-fat milk for 1 h. Membranes were incubated with primary antibodies overnight at +4°C, followed by incubation with horseradish peroxidase-conjugate secondary antibodies (Bio-Rad) and revealed with western chemoluminescent HRP substrate (Millipore, Immobilon WBKLS0500). Chemoluminescent signals were acquired with the iBright CL1000 Imaging System (Thermo Fisher, United States). Quantitative analysis was performed using ImageJ software. Primary antibodies used were rabbit anti-p62 (MBL International Corporation, United States, PM045), mouse anti-COXII (Abcam, United Kingdom, ab110258), mouse anti-COXIV (Abcam, United Kingdom, ab33985), rabbit anti-LC3 A/B (Cell Signaling, United States, D3U4C, 12741S), rabbit anti-HSP90 (Cell Signalling; United States, E289, 4875), and mouse anti-vinculin (Santa Cruz Biotechnology, United States, 7F9, sc-73614).

#### Mouse neural progenitor cell-derived neurosphere generation and treatment

Cryopreserved mouse neural progenitor cells were cultured at 37°C and 5% CO_2_ as a suspension culture in a proliferation medium consisting of Dulbecco’s modified Eagle medium (DMEM) supplemented with B27 (Invitrogen GmbH, Karlsruhe, Germany). When spheres reached 0.5 mm in diameter, they were chopped up to passage 3 with a McIlwain tissue chopper. Differentiation was initiated by growth factor withdrawal in a differentiation medium supplemented with N2 (insulin, transferrin, sodium selenite, putrescine, and progesterone; Invitrogen), plated onto poly-d-lysine/laminin–coated chamber slides (BD Bioscience, Erembodegem, Belgium). Cobalt chloride, deferoxamine, and 2,2′-dipyridyl were added at the desired concentration directly into the differentiation medium. The rate of neuronal differentiation and cell death were detected after 48 or 72 h of drug treatment.

Afterward, cells were fixed with 4% PFA (Sigma-Aldrich, Germany) for 30 min at 37°C and washed with PBS. Cells were stained with the primary antibody mouse-anti-βIII-tubulin for 1 h at 37°C. After washing with PBS, cells were incubated with the secondary antibody antimouse-Alexa-448 (Invitrogen, United States) for 30 min at 37°C. Nuclei were stained with Hoechst 33258 (Sigma-Aldrich, Germany). Samples were analyzed using a fluorescent microscope (Carl Zeiss, Germany) and AxioVision Rel.4.8 software (Carl Zeiss, Germany).

For the quantification of neuronal differentiation, the number of βIII-tubulin + cells in a given area was manually counted and divided by the total number of nuclei of the same area, which were counted automatically with ImageJ, and the result was expressed in percentage. Afterward, the values obtained for all exposed groups were expressed in percentage of the value of the control of the corresponding experiment. Cell death (necrosis) quantification was performed by counting cells stained with propidium iodide normalized to the total number of cells stained with Hoechst.

## Data Availability

The raw data supporting the conclusions of this article will be made available by the authors, without undue reservation.

## Supplementary material

Supplementary Figure S1
**(A,B)** Representative pictures **(A)** and quantification **(B)** of the poly-Q40 reporter strain AM141: rmIs133 [*unc-54p*::Q40::YFP] in 4-, 7-, and 10-days-old adult worms left untreated (−) or treated from eggs with CoCl_2_ 500 µM (+). The number of poly-Q40 aggregates was counted using the “analyze particles…” tools in ImageJ after thresholding and converting the acquired images (right side of panel A) into a binary form (left side of panel A). (B) Bar graph represents means ± SD (*n* = 3, N=10–15). Comparison between untreated and CoCl_2_ at the same time point was performed using the unpaired t-test. **p* value < 0.05 and **** *p* value < 0.0001. Scale bar = 100 µm.

Supplementary Figure S2 Developmental rate of wild-type (WT) and *hif-1* KO animals from eggs through four different larval stages, until young (YA) and gravid (GA) adults, either left untreated (−) or treated from eggs with 500 µM CoCl_2_ (+) and followed for 5 days after hatching at 20°C.

Supplementary Figure S3
**(A–D)** Western blot analysis of LC3II **(A)**, p62 **(B)**, COXII **(C)**, and COXIV **(D)** proteins extracted from HeLa cells left untreated (−) or treated (+) with 250 µM alone or in combination with 1mM of CoCl_2_ at different time points (4, 16, and 24 hours). Proteins were normalized using vinculin. Representative Western blots are shown. Bar graph represents means ± SD (N = 3). Comparison between groups was performed using the unpaired t-test. **p* value < 0.05.

Supplementary Figure S4 Representative pictures of HeLa cells left untreated (−) or treated (+) with 250 µM alone or in combination with 1 mM of CoCl_2_ (+) at different time points (4, 16, and 24 hours). White squares represent the magnified crop (2x) shown on the right. Scale bar = 100 µm.
