# Supplementary figures and images for: Cobalt chloride has beneficial effects across species through a hormetic mechanism
Source: Front Cell Dev Biol. 2022 Oct 25;10:986835. doi: 10.3389/fcell.2022.986835 (PMC9642780; doi:10.3389/fcell.2022.986835)

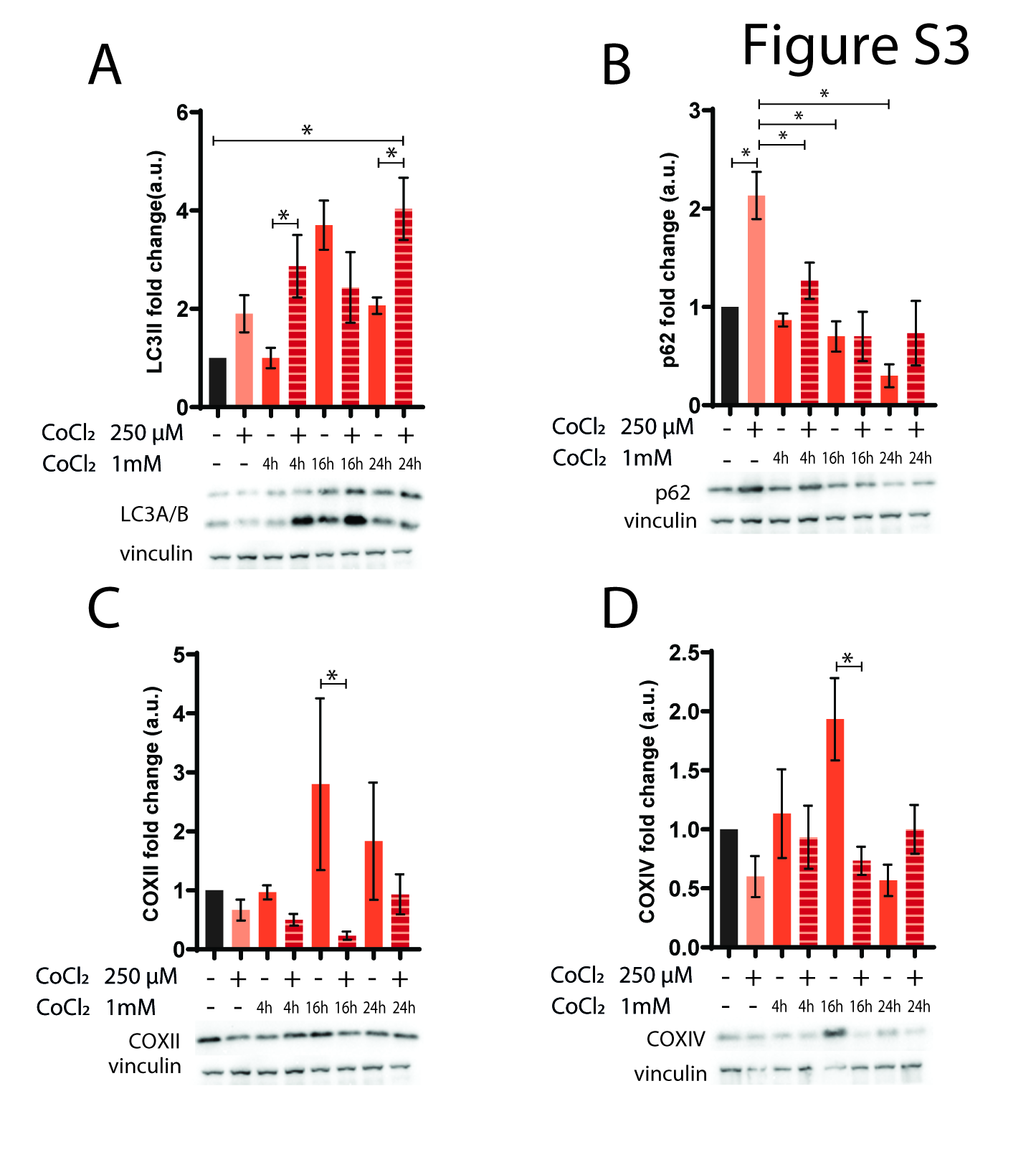

Supplement: Supplementary file 1 [file Image3.tif]

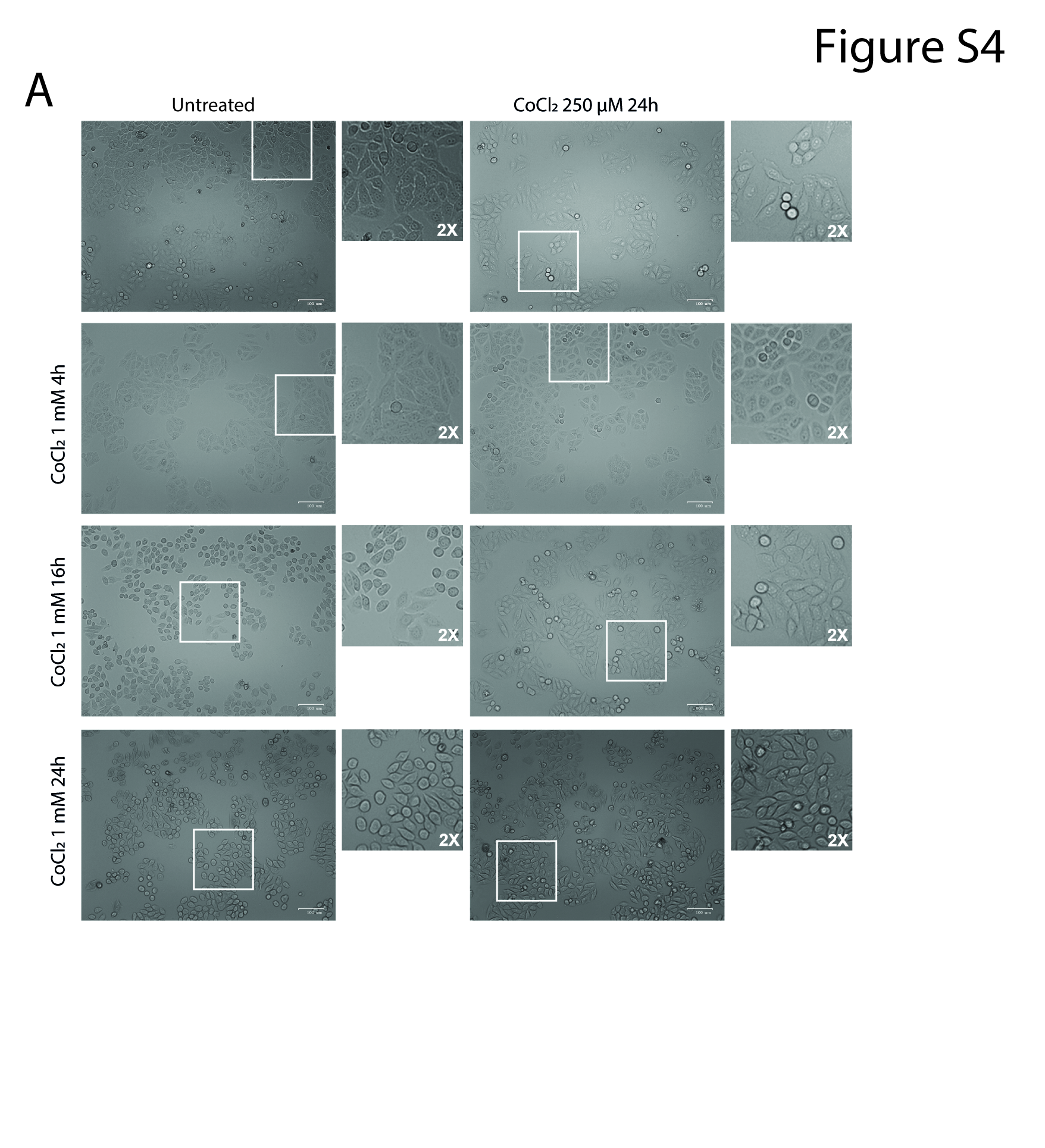

Supplement: Supplementary file 2 [file Image4.tif]

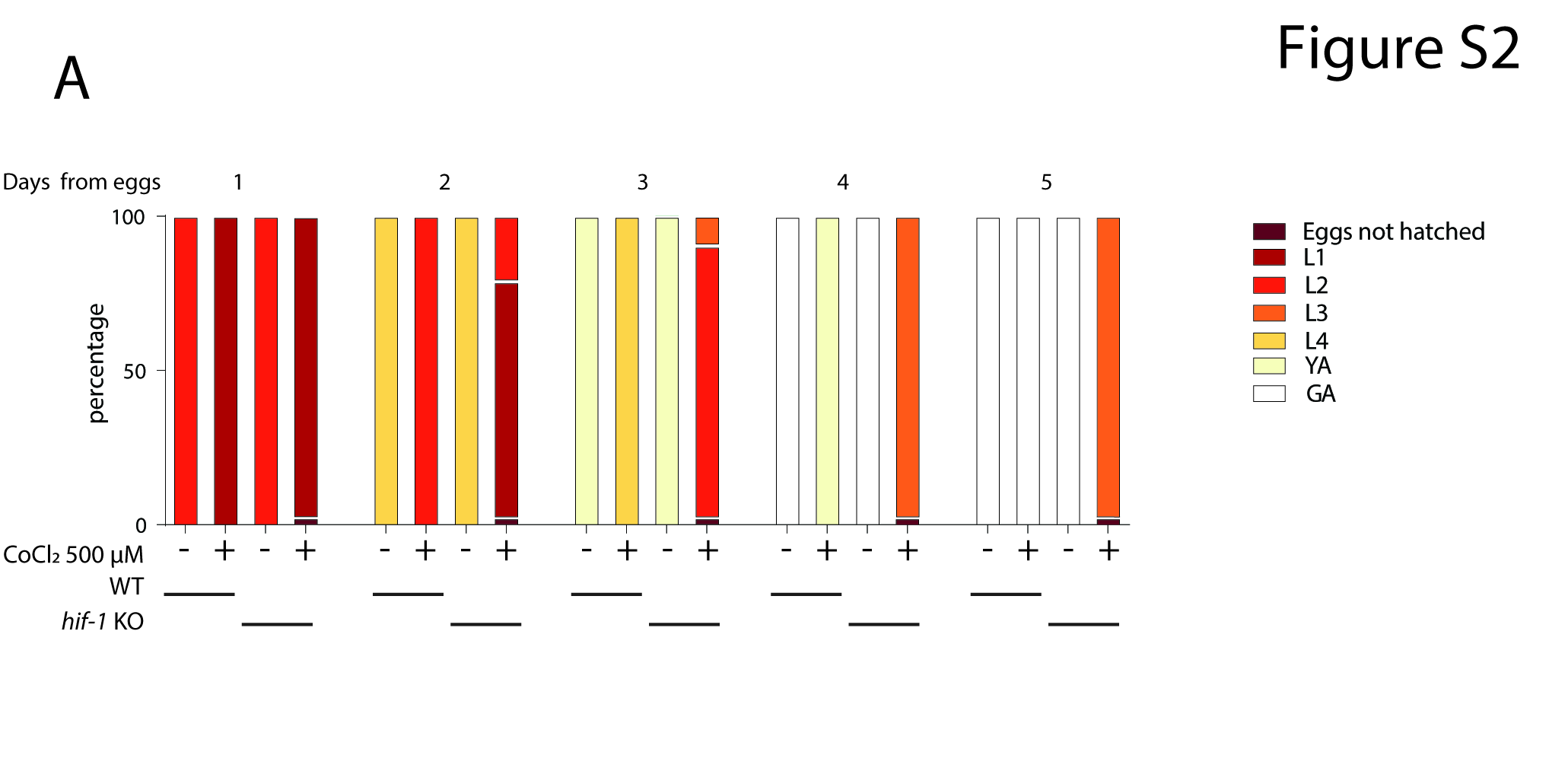

Supplement: Supplementary file 3 [file Image2.tif]

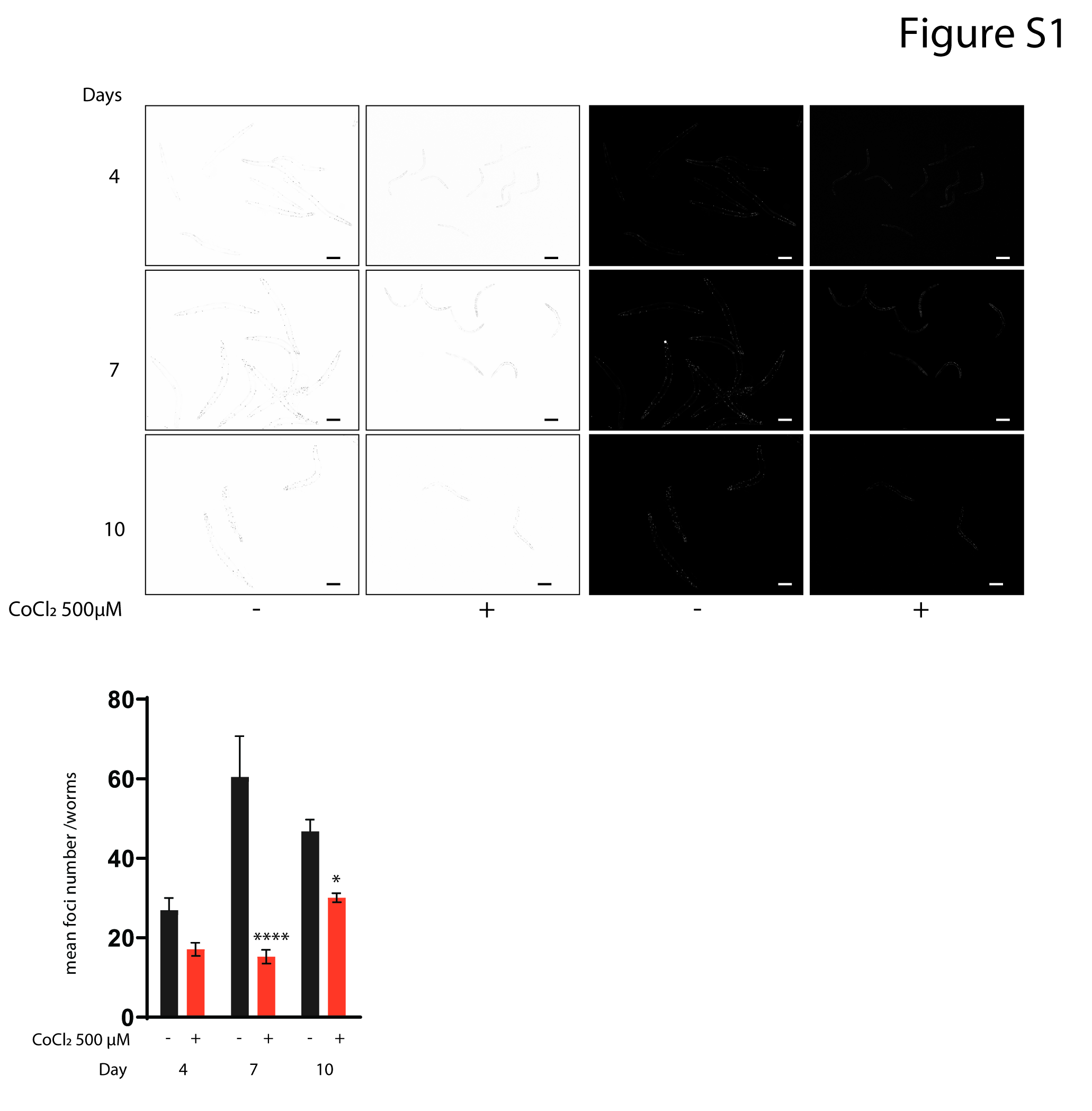

Supplement: Supplementary file 4 [file Image1.tif]
